# Angiotensin I and angiotensin II concentrations and their ratio in catecholamine-resistant vasodilatory shock

**DOI:** 10.1186/s13054-020-2733-x

**Published:** 2020-02-06

**Authors:** Rinaldo Bellomo, Richard G. Wunderink, Harold Szerlip, Shane W. English, Laurence W. Busse, Adam M. Deane, Ashish K. Khanna, Michael T. McCurdy, Marlies Ostermann, Paul J. Young, Damian R. Handisides, Lakhmir S. Chawla, George F. Tidmarsh, Timothy E. Albertson

**Affiliations:** 1 Centre for Integrated Critical Care, Department of Medicine & Radiology, The University of Melbourne, Melbourne, Australia; 2 Australian and New Zealand Intensive Care Research Centre, School of Public Health and Preventive Medicine, Monash University, Melbourne, Australia; 3 Department of Medicine, Pulmonary and Critical Care Division, Northwestern University Feinberg School of Medicine, Chicago, IL USA; 4 Department of Medicine, Division of Nephrology, Baylor University Medical Center, Dallas, TX USA; 5 Clinical Epidemiology Program, Ottawa Hospital Research Institute, Ottawa, Ontario Canada; 6 Department of Medicine (Critical Care), University of Ottawa, Ottawa, Ontario Canada; 7 School of Epidemiology and Public Health, University of Ottawa, Ottawa, Ontario Canada; 8 Department of Medicine, Division of Pulmonary, Allergy, Critical Care and Sleep Medicine, Emory University, Atlanta, GA USA; 9 Department of Medicine and Radiology, Royal Melbourne Hospital, The University of Melbourne, Melbourne Medical School, Parkville, Australia; 10 Department of Anesthesiology, Section on Critical Care Medicine, Wake Forest University School of Medicine, Winston-Salem, NC USA; 11 Outcomes Research Consortium, Cleveland, OH USA; 12 Division of Pulmonary & Critical Care Medicine, University of Maryland School of Medicine, Baltimore, MD USA; 13 Department of Critical Care, King’s College London, Guy’s & St Thomas’ Hospital, London, UK; 14 Medical Research Institute of New Zealand, Wellington, New Zealand; 15 Intensive Care Unit, Wellington Hospital, Wellington, New Zealand; 16 La Jolla Pharmaceutical Company, San Diego, CA USA; 17 Stanford University School of Medicine, Palo Alto, CA USA; 18 Department of Internal Medicine, Division of Pulmonary, Critical Care and Sleep Medicine, School of Medicine, University of California, Davis, Sacramento, CA USA; 19 Department of Veterans Affairs, Northern California Health System, Mather, CA USA

**Keywords:** Angiotensin I, Angiotensin II, ACE, ACE dysfunction, Sepsis, Vasodilatory shock

## Abstract

**Background:**

In patients with vasodilatory shock, plasma concentrations of angiotensin I (ANG I) and II (ANG II) and their ratio may reflect differences in the response to severe vasodilation, provide novel insights into its biology, and predict clinical outcomes. The objective of these protocol prespecified and subsequent post hoc analyses was to assess the epidemiology and outcome associations of plasma ANG I and ANG II levels and their ratio in patients with catecholamine-resistant vasodilatory shock (CRVS) enrolled in the Angiotensin II for the Treatment of High-Output Shock (ATHOS-3) study.

**Methods:**

We measured ANG I and ANG II levels at baseline, calculated their ratio, and compared these results to values from healthy volunteers (controls). We dichotomized patients according to the median ANG I/II ratio (1.63) and compared demographics, clinical characteristics, and clinical outcomes. We constructed a Cox proportional hazards model to test the independent association of ANG I, ANG II, and their ratio with clinical outcomes.

**Results:**

Median baseline ANG I level (253 pg/mL [interquartile range (IQR) 72.30–676.00 pg/mL] vs 42 pg/mL [IQR 30.46–87.34 pg/mL] in controls; *P* <  0.0001) and median ANG I/II ratio (1.63 [IQR 0.98–5.25] vs 0.4 [IQR 0.28–0.64] in controls; *P* <  0.0001) were elevated, whereas median ANG II levels were similar (84 pg/mL [IQR 23.85–299.50 pg/mL] vs 97 pg/mL [IQR 35.27–181.01 pg/mL] in controls; *P* = 0.9895). At baseline, patients with a ratio above the median (≥1.63) had higher ANG I levels (*P* <  0.0001), lower ANG II levels (*P* <  0.0001), higher albumin concentrations (*P* = 0.007), and greater incidence of recent (within 1 week) exposure to angiotensin-converting enzyme inhibitors (*P* <  0.00001), and they received a higher norepinephrine-equivalent dose (*P* = 0.003). In the placebo group, a baseline ANG I/II ratio <1.63 was associated with improved survival (hazard ratio 0.56; 95% confidence interval 0.36–0.88; *P* = 0.01) on unadjusted analyses.

**Conclusions:**

Patients with CRVS have elevated ANG I levels and ANG I/II ratios compared with healthy controls. In such patients, a high ANG I/II ratio is associated with greater norepinephrine requirements and is an independent predictor of mortality, thus providing a biological rationale for interventions aimed at its correction.

**Trial registration:**

ClinicalTrials.gov identifier NCT02338843. Registered 14 January 2015.

## Background

Vasodilatory shock, a form of life-threatening generalized acute circulatory failure [1, 2], affects many patients in intensive care [3] and is associated with high mortality [4]. Vasodilatory shock has many etiologies, including but not limited to sepsis (the most common cause), inflammatory shock without infection (e.g., pancreatitis), postsurgical vasoplegia, endocrine shock, and spinal shock [5]. The primary goal of the hemodynamic treatment of such patients is to restore adequate mean arterial pressure (MAP) [6] with fluid resuscitation and/or vasopressors [7–9]. However, some patients are resistant to vasopressor therapy and require high doses to reach target MAP. This catecholamine-resistant vasodilatory shock (CRVS) is associated with adverse events [10, 11] and high mortality rates [12–14], but its pathophysiology is not well understood.

The peptide angiotensin I (ANG I) is an integral part of the renin-angiotensin-aldosterone system, which regulates blood pressure and is converted by the angiotensin-converting enzyme (ACE) to ANG II, making the ANG I/II ratio a marker of ACE function [15, 16]. Low levels of ANG II, a potent vasoconstrictor, are associated with increased mortality in severe sepsis [17], vasodilatory shock [18], and acute respiratory distress syndrome [19], all of which are conditions with endothelial injury, decreased endothelium-bound ACE activity, and decreased capacity to convert ANG I to ANG II [18–20]. Thus, the ANG I/II ratio may be elevated in CRVS and predict worse clinical outcomes. These considerations have become increasingly relevant since synthetic human ANG II was approved in the USA and Europe to increase MAP in patients with vasodilatory shock [21].

Accordingly, as part of the randomized, double-blind, phase 3 ATHOS-3 (Angiotensin II for the Treatment of High-Output Shock) trial (ClinicalTrials.gov, NCT02338843), we measured ANG I and II levels of patients with CRVS before initiation of synthetic human ANG II infusion and calculated their ratio. We hypothesized that such patients would have elevated ANG I levels and an increased ANG I/II ratio compared with healthy controls and that a higher ANG I/II ratio would be associated with increased norepinephrine requirements at baseline and with increased mortality.

## Methods

### Patients

#### Patients with vasodilatory shock

The ATHOS-3 study protocol, including patient characteristics, has been previously published [22, 23]. In brief, patients with catecholamine-resistant hypotension (defined as those with a total vasopressor dose >0.2 mcg/kg/min for ≥6 h) and high-output shock (defined as central venous oxygen saturation >70% with central venous pressure >8 mmHg or cardiac index >2.3 L/min/m^2^) were randomized and treated with either ANG II or placebo, plus standard of care. Blood samples were drawn and stored after randomization and prior to administration of study drug. Collected blood was centrifuged (2000 g for 10 min) and stored at –80 °C until shipped for analysis.

#### Healthy control sera

As part of the ANG I and ANG II assay validation, ANG I and ANG II levels were measured in banked sera donated by healthy volunteers.

#### ANG I and ANG II assessments

Endogenous serum concentrations of ANG I and ANG II were measured by ultra-performance liquid chromatography with tandem mass spectrometry detection, capable of measuring angiotensin peptide levels as low as 10 pg/mL (inVentiv Health Clinique, Quebec City, Quebec, Canada). Following rapid thawing of the serum, samples were stabilized with a combination of aliskiren, pepstatin A, and o-phenanthroline in acidified dimethyl sulfoxide combined with a mixture of EDTA and 4-(hydroxymercury) benzoic acid in phosphate-buffered saline. All samples were spiked with stable-isotope-labeled internal standards for ANG I and ANG II at a concentration of 50 pg/mL. Following protein precipitation using acetonitrile with 1% formic acid and solid-phase extraction (Oasis MCX; Waters Corporation, Milford, MA, USA) of the supernatant, samples underwent liquid chromatography-tandem mass spectrometry analysis using a reverse-phase analytical column (Acquity CSH C18; Waters Corporation) operating in line with an XEVO TQ-S triple quadrupole mass spectrometer (Waters Corporation) in multiple reaction monitoring. The sum of the signal from three different mass transitions per peptide was measured, and angiotensin concentrations were calculated by relating the ratio of peptide signal to internal standard signal.

### Statistical analyses

Analyses of baseline ANG I, ANG II, and ANG I/II ratio and association with survival were prespecified. All other analyses, including comparison to healthy controls, were post hoc. Wilcoxon rank-sum test, Fisher’s exact test for binary outcomes, and chi-square statistic for other categorical outcomes were used for comparisons. Survival from the time of randomization to time of death from any cause was analyzed by the Kaplan–Meier formula. Estimates and confidence intervals were calculated by the product limit method and Greenwood’s formula for the variance and included the difference between treatment arms. For missing data in time-to-event analyses, including mortality at day 28, censored data techniques were utilized. Patients with missing data were censored on the last known survival date up to the specified endpoint (i.e., day 28).

Differences in survival between ANG I/II ratios above and below the median were analyzed by a two-sided log-rank test for mortality to day 28. Multivariate analyses were conducted for mortality to day 28, which included a stratified log-rank test using baseline strata and covariates that were not balanced. To adjust for the impact of multiple comparisons, a *P* <  0.01 was used to infer statistical significance.

## Results

We studied 321 patients with vasodilatory shock. Sera from 24 healthy subjects formed the control group. Baseline ANG I and II levels are summarized in Table 1. In comparison to healthy controls, vasodilatory shock patients had substantially (roughly 6-fold) higher ANG I levels (253 pg/mL [interquartile range (IQR) 72.30–676.00 pg/mL] vs 42 pg/mL [IQR 30.46–87.34 pg/mL]; difference *P* <  0.0001) and higher ANG I/II ratios (1.63 [IQR 0.98–5.25] vs 0.4 [IQR 0.28–0.64]; difference *P* <  0.0001). In contrast, ANG II levels were not different between groups (84 pg/mL [IQR 23.85–299.50 pg/mL] vs 97 pg/mL [IQR 35.27–181.01 pg/mL]; difference *P* = 0.9895). Distribution of baseline ANG I and II levels and ANG I/II ratio for vasodilatory shock patients can be found in Additional file 1: Figures S1–S3 (Table 1).

**Table 1:** Baseline angiotensin I, angiotensin II, and angiotensin I/II ratio in ATHOS-3 patients and healthy controls

	Angiotensin I^a^	Angiotensin II^a^	Angiotensin I/II ratio
ATHOS-3			
Number of patients	321	321	321
Number with data	286	284	281
Mean (SD)	589 (942)	276 (488)	10.3 (27.6)
Median	253^b^	84^c^	1.63^b^
IQR	72.30–676.00	23.85–299.50	0.98–5.25
Healthy controls			
Number of patients	24	24	24
Number with data	24	24	24
Mean (SD)	63 (57)	123 (100)	1.59 (3.3)
Median	42	97	0.39
IQR	30.46–87.34	35.27–181.01	0.28–0.64

*ATHOS-3* Angiotensin II for the Treatment of High-Output Shock, *IQR* interquartile range, *SD* standard deviation

^a^All values in pg/mL. Values are rounded to nearest integer except for ratio

^b^*P* < 0.0001 compared with healthy controls

^c^*P* = 0.9895 compared with healthy controls

### Angiotensin I/II ratio

The median ANG I/II ratio across treatment arms at baseline was 1.63 (IQR 0.98–5.25). Patient demographics and disease characteristics by baseline median ANG I/II ratio were largely similar between groups (Table 2). However, recent exposure to ACE inhibitors was significantly more common in patients with a ratio above the median. Moreover, patients with a higher ANG I/II ratio had higher serum albumin concentrations and were receiving a higher dose of vasopressor support (norepinephrine-equivalent dose) at baseline. Baseline ANG I/II ratios were similar between the placebo (*n* = 139) and ANG II treatment arms (*n* = 142) (Table 2).

**Table 2:** Baseline demographics and disease characteristics for patients with vasodilatory shock

	Baseline angiotensin I/II ratio		*P* value
	<1.63 (*n* = 141)	≥1.63 (*n* = 140)	
Age, years			
Median (IQR)	65 (51–76)	63 (53–75)	0.522
Sex (male/female), %	58.9%/41.1%	61.4%/38.6%	0.715
Baseline MAP, mmHg			
Median (IQR)	66.3 (63.3–68.7)	67 (63.7–68.7)	0.891
APACHE II			
Median (IQR)	27 (22–33)	29 (23–34)	0.112
Albumin (g/dL)			
Median (IQR)	2.2 (1.7–2.7)	2.4 (2.0–2.8)	0.007
SCVO_2_, %			
Median (IQR)	77 (73.0–83.0)	76.5 (72.2–82.0)	0.211
Central venous pressure (mmHg)			
Median (IQR)	12 (10–15)	12 (10–16)	0.317
Cardiac index			
Median (IQR)	3.1 (2.6–4.0)	3.1 (2.8–3.7)	0.796
MELD score			
Median (IQR)	22 (15–25)	23 (17–28)	0.046
Chest X-ray finding of ARDS, *n* (%)	44 (31.2%)	33 (23.7%)	0.182
Medical history of ARDS, *n* (%)	33 (23.4%)	15 (10.7%)	0.007
Exposure to ACE inhibitors, *n* (%)	1 (0.7%)	27 (19.3%)	< 0.001
Exposure to ARBs, *n* (%)	13 (9.2%)	7 (5.0%)	0.246
AKI with dialysis/CRRT, *n* (%)	39 (27.7%)	52 (37.1%)	0.098
Vasopressin use during 6 h before randomization, *n* (%)	93 (66.0%)	102 (72.9%)	0.244
Baseline norepinephrine-equivalent dose (μg/kg/min)			
Median (IQR)	0.30 (0.22–0.49)	0.39 (0.24–0.59)	0.006
Median (IQR) ANG I level, (pg/mL)	134 (42.7–468)	354.5 (129–869.5)	< 0.001
Median (IQR) ANG II level, (pg/mL)	164 (45–552)	42.35 (11.5–134.5)	< 0.001
Median ANG I/II ratio (IQR)	0.98 (0.67–1.21)	5.36 (2.64–14.73)	< 0.001

*ACE* angiotensin-converting enzyme, *AKI* acute kidney injury, *ANG* angiotensin, *APACHE II* Acute Physiology and Chronic Health Evaluation II, *ARB* angiotensin II receptor type I blocker, *ARDS* acute respiratory distress syndrome, *CRRT* continuous renal replacement therapy, *IQR* interquartile range, *MAP* mean arterial pressure, *MELD* model for end-stage liver disease, *SCVO*_*2*_ central venous oxygen saturation

### Survival by baseline ANG I/II ratio

**Fig. 1:**
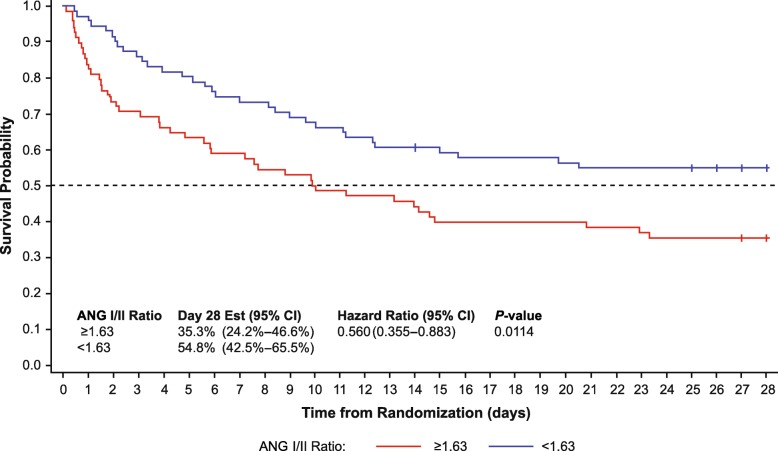
Survival to day 28 by baseline ratio of angiotensin I/II (<1.63 or ≥1.63, the population median). *ANG* angiotensin, *CI* confidence interval, *Est* estimate

Mortality in the trial’s placebo treatment arm was 64.7% in those with baseline ANG I/II ratio above the median and 45.2% in those with a ratio below the median (Fig. 1). In a multivariate analysis of mortality in the placebo arm, the baseline ANG I/II ratio was a significant predictor of overall mortality (hazard ratio 0.54; *P* = 0.0111) (Table 3, Fig. 1)

**Table 3:** Multivariate analyses of survival in placebo treatment arm

Characteristic	Hazard ratio (95% CI)	*P* value
Full model		
Baseline ANG I/II ratio	0.52 (0.30–0.89)	0.0180
Age ≥65 years	1.18 (0.73–1.90)	0.4925
Gender, male	1.04 (0.62–1.75)	0.8710
Baseline albumin <2.5 g/dL	1.38 (0.83–2.28)	0.2179
Baseline MAP <65 mmHg	1.87 (1.14–3.07)	0.0125
Baseline APACHE II score >30	1.63 (0.98–2.71)	0.0620
Exposure to ACEI, yes	0.34 (0.11–1.03)	0.0554
Baseline NE equivalent dose ≥0.5 μg/kg/min	1.59 (0.95–2.65)	0.0772
Medical history of ARDS, yes	1.16 (0.62–2.16)	0.6485
Baseline ANG I <253 pg/mL	0.54 (0.27–1.12)	0.0968
Baseline ANG II <83.75 pg/mL	1.47 (0.70–3.09)	0.3049

*ACEI* angiotensin-converting enzyme inhibitor, *APACHE II* Acute Physiology and Chronic Health Evaluation II, *ANG* angiotensin, *ARDS* acute respiratory distress syndrome, *CI* confidence interval, *MAP* mean arterial pressure, *NE* norepinephrine

## Discussion

We measured the plasma concentrations of ANG I and ANG II and calculated their ratio at baseline in patients enrolled in the ATHOS-3 study. We found that, in patients with CRVS, ANG I levels were higher than in healthy controls. We also found that despite much higher ANG I concentrations in the ATHOS-3 patients, ANG II levels were similar to those in healthy controls; this led to increased ANG I/II ratios. These observations suggest that ACE function and the conversion of ANG I to ANG II may be disordered in vasodilatory shock. Moreover, we found that ANG I/II ratios above the median were associated with specific baseline features (i.e., recent use of ACE inhibitor, greater dose of norepinephrine-equivalent administration, and greater severity of illness). Finally, we found that a high ANG I/II ratio predicted increased mortality.

### Relationship to previous studies

Previous studies have reported that the baseline ANG I/II ratio averaged 0.38 in otherwise healthy patients with hypertension [15]; this is consistent with the ratio of 0.4 in healthy sera measured. The median ratio value of 1.63 for patients in the present study suggests a possible pathological decrease in conversion of ANG I to ANG II in patients with CRVS. Endothelial injury is common during septic shock. Thus, endothelial membrane–bound ACE activity may be reduced during shock. Logically, reduced ACE activity should lead to decreased ANG I to ANG II conversion and an increased ratio. A significant proportion of ATHOS-3 patients had high ANG I/II ratios, suggesting decreased ACE activity. Low levels of ANG II and ACE activity on day 1 have been previously reported in patients with sepsis and appear associated with a poor prognosis [17]. Decreased ACE activity could be due to an intrinsic defect in ACE function [20] or to small peptides with ACE inhibitory properties [24]. In addition, at least two pro-inflammatory cytokines (tumor necrosis factor-α [TNF-α] and interleukin-1β) downregulate ACE in cultured human endothelial cells [25]. Finally, while not examined in this study, different single-nucleotide polymorphisms of ACE can affect ACE activity and are associated with mortality rates in septic shock [26], possibly through interactions between TNF-α and such polymorphisms [27]. It appears biologically plausible that a high ANG I/II ratio may reflect decreased ACE activity. In keeping with this notion, the recent use of ACE inhibitors was markedly more common in patients with a high ANG I/II ratio in our study.

Another key enzyme, ACE2, can also affect the ANG I/II ratio. ACE2 catalyzes the conversion of ANG II to ANG (1–7) [28], and increased ACE2 activity may also decrease ANG II levels and increase ANG I/II ratios. Therefore, high ACE2 activity may contribute to a high ANG I/II ratio in vasodilatory shock.

### Study implications

Our findings suggest that in many patients with CRVS, there is an imbalance between ANG I and ANG II levels. This imbalance may be related to changes in ACE1 and/or ACE2 activity, which may relatively diminish ANG II generation and can be exacerbated by recent ACE inhibitor administration. Moreover, the findings imply that diminished ability to convert ANG I to ANG II may contribute to a catecholamine-resistant vasodilatory state and increase the risk of death. In their aggregate, these findings suggest that there is a biological rationale for the exogenous administration of ANG II in CRVS.

### Strengths and limitations

To our knowledge, this is one of the first studies to evaluate serum ANG I and ANG II levels and the ANG I/II ratio in patients with CRVS. Only a single recent pilot study found that increased ANG I levels were correlated with mortality [29]. In comparison, our study was much larger and involved several hundred patients in multiple countries and continents, thus providing a high level of external validity. In addition, this study utilized a double-blind, placebo-controlled, phase 3 registration trial design, assuring that characteristics and outcomes were collected prospectively and were independently monitored; this minimized selection and ascertainment bias. The measurements of ANG I and ANG II were performed by an independent laboratory blinded to clinical characteristics, thus further minimizing bias. Moreover, the analysis of such data followed a prespecified protocol. Finally, the associations observed appear logical and consistent with current knowledge of the physiology and pathophysiology of ANG I, ANG II, and ACE1 and ACE2 activity in inflammatory states.

Our study had limitations. We dichotomized ANG I/II ratios as part of our assessment. Such an approach simplifies comparisons but is insensitive to the continuous nature of biological variables. Thus, the correct specific cutoff point to inform clinical decisions remains unknown. Follow-up was to 28 days only, so implications for longer survival windows could not be made. In addition, ACE activity was not measured directly; rather, ACE activity was inferred from the ratio of ANG I/II in this study. However, patients with prior exposure to ACE inhibitors appeared to be particularly prone to a high baseline ANG I/II ratio, indicating that, in at least some patients, a high baseline ratio very likely resulted from decreased ACE activity. We did not measure the ANG I/II ratio in real time. However, ANG I and II concentrations were collected prospectively as part of a prespecified analysis. We did not measure ACE2 activity as part of the ATHOS-3 study. Thus, our suggestion that increased ACE2 activity may affect the ANG I/II ratio remains speculative. Further studies will require a more detailed assessment of the increasingly complex angiotensin family of molecules and their interaction with ACE1 and ACE2 activity.

## Conclusions

In CRVS, both ANG I and the ANG I/II ratio are elevated. High ANG I/II ratios are associated with specific baseline clinical features and predict increased mortality. These observations provide a biological rationale for interventions aimed at correcting such imbalance.

## Supplementary information

**Additional file 1: Figure S1.** Angiotensin I distribution at baseline. **Figure S2.** Angiotensin II distribution at baseline. **Figure S3.** Angiotensin I/II ratio distribution at baseline.

## Data Availability

The data that support the findings of this study are available from La Jolla Pharmaceutical Company, but restrictions apply to the availability of these data, which were used under license for the current study and so are not publicly available. However, data are available from the authors upon reasonable request and with the permission of La Jolla Pharmaceutical Company.

## Abbreviations

ACE: Angiotensin-converting enzyme
ANG: Angiotensin
ATHOS-3: Angiotensin II for the Treatment of High-Output Shock
CRVS: Catecholamine-resistant vasodilatory shock
MAP: Mean arterial pressure
TNF-α: Tumor necrosis factor-α
