# Supplementary material for: Angiotensin I and angiotensin II concentrations and their ratio in catecholamine-resistant vasodilatory shock
Source: Crit Care. 2020 Feb 6;24:43. doi: 10.1186/s13054-020-2733-x (PMC7006163; doi:10.1186/s13054-020-2733-x)
Supplement: Supplementary file 1 — Additional file 1: Figure S1. Angiotensin I distribution at baseline. Figure S2. Angiotensin II distribution at baseline. Figure S3. Angiotensin I/II ratio distribution at baseline. [file 13054_2020_2733_MOESM1_ESM.docx]

**SUPPLEMENTAL MATERIAL**

**Figure S1**. Angiotensin I distribution at baseline


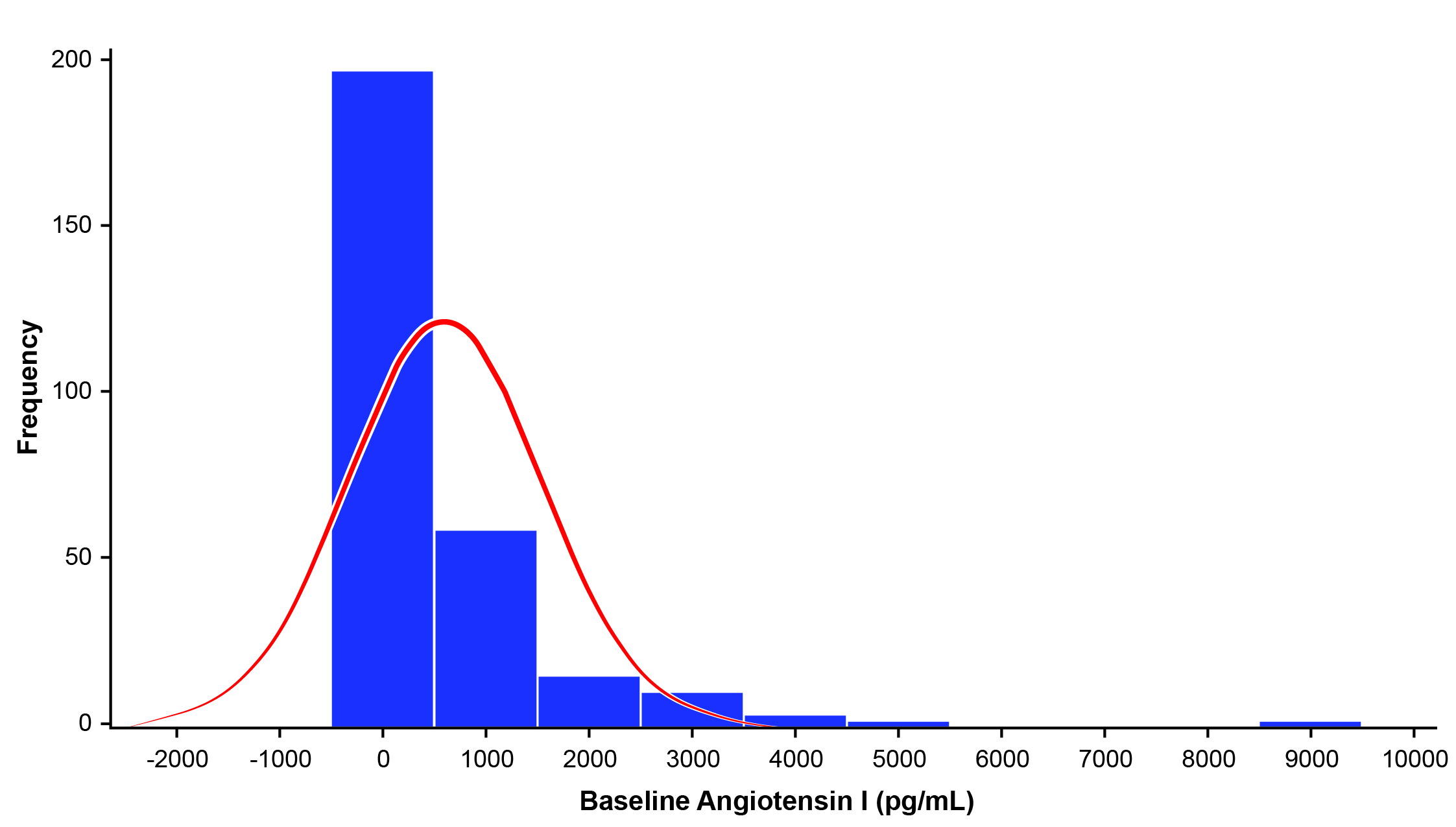


Figure includes all patients who had a sample measurement for Angiotensin I.

**Figure S2.** Angiotensin II distribution at baseline.


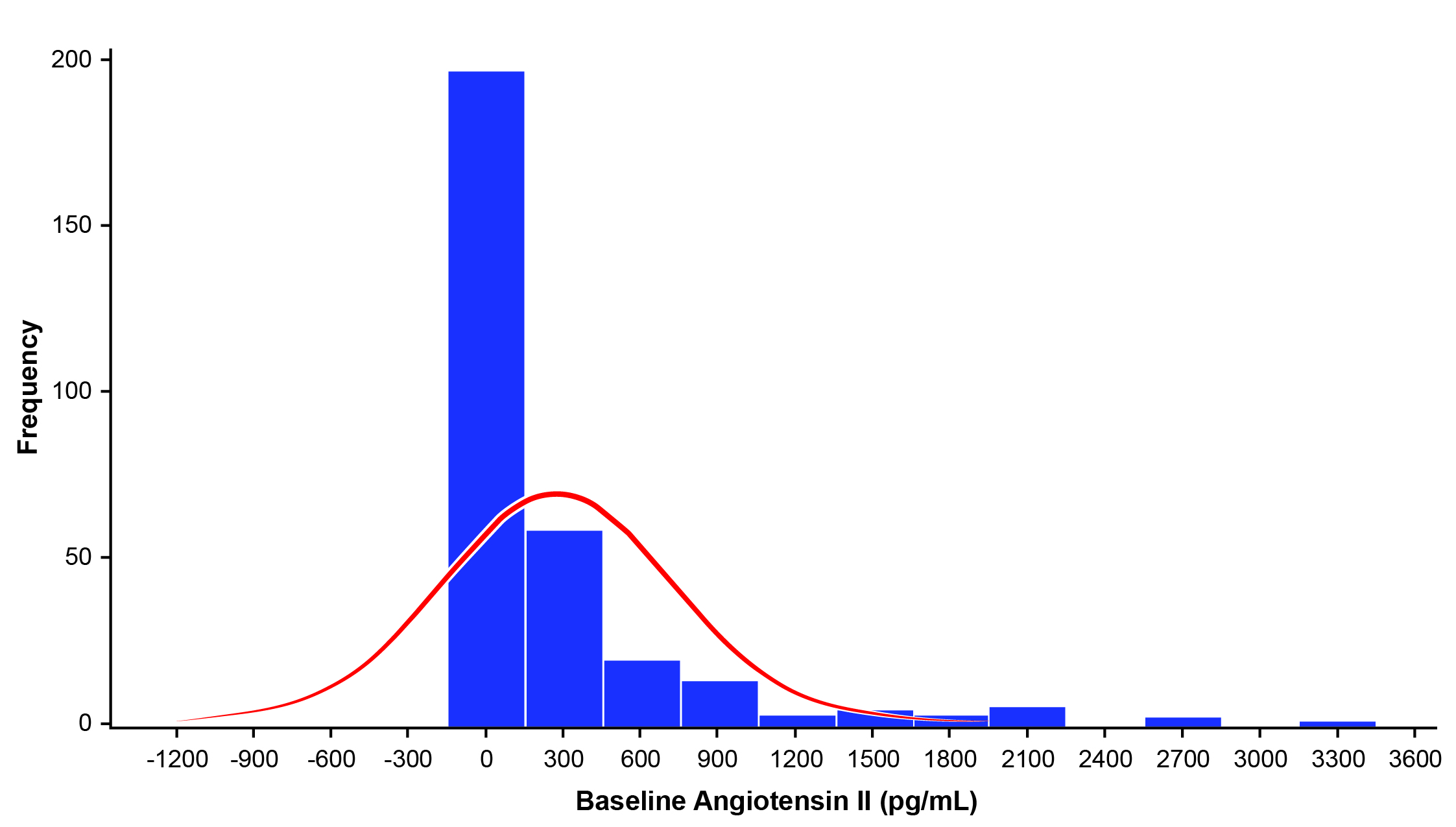


Figure includes all patients who had a sample measurement for Angiotensin II.

**Figure S3**. Angiotensin I/II ratio distribution at baseline.


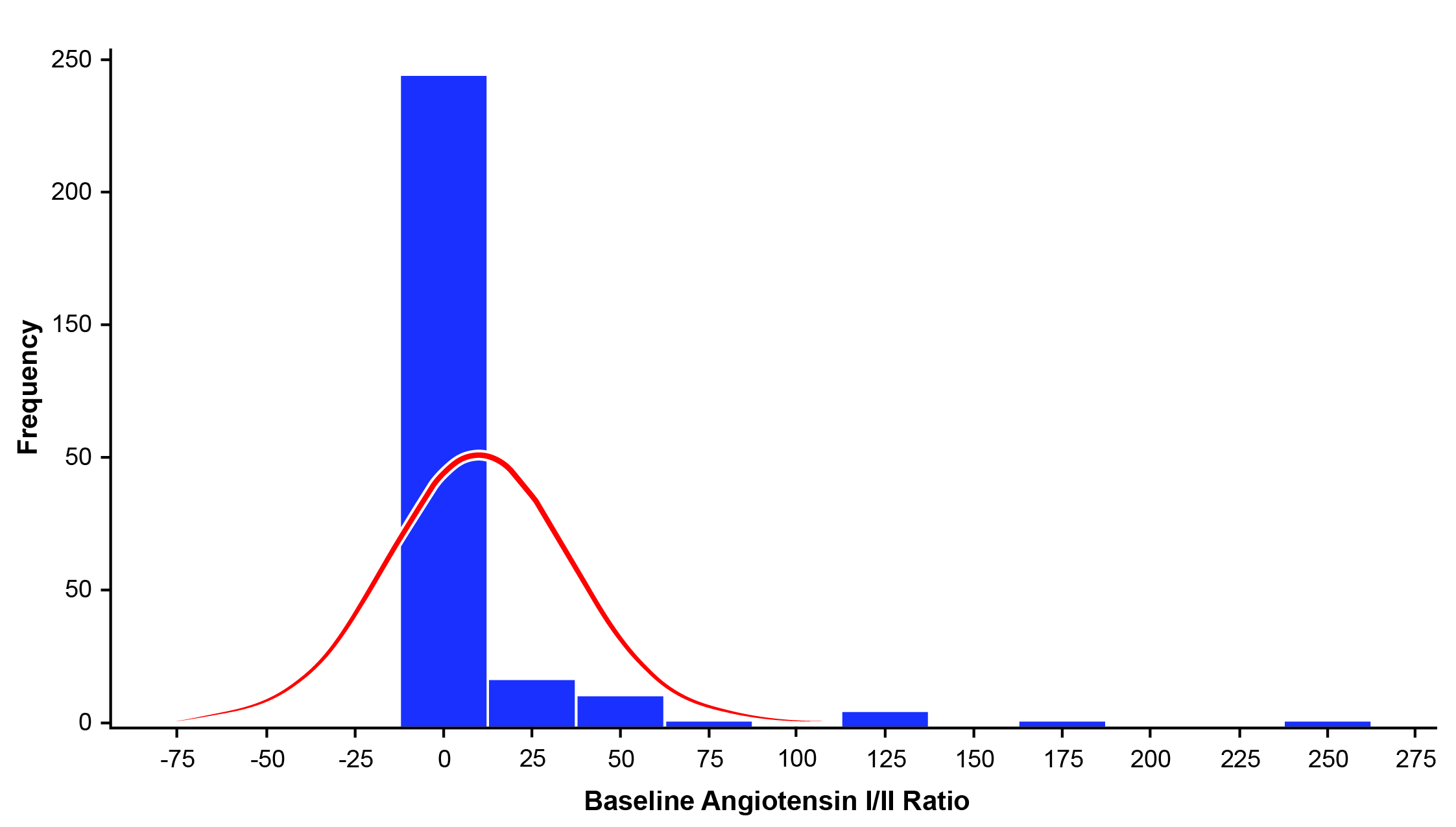


Figure includes all patients who had a calculated Ang I/II ratio.
